# Uptake of COVID-19 vaccination among community-dwelling individuals receiving healthcare for substance use disorder and major mental illness: a matched retrospective cohort study

**DOI:** 10.3389/fpubh.2024.1426152

**Published:** 2024-07-05

**Authors:** Lucie Richard, Anna Holland, Vivian Aghanya, Michael A. Campitelli, Stephen W. Hwang

**Affiliations:** ^1^MAP Centre for Urban Health Solutions, Unity Health Toronto, Toronto, ON, Canada; ^2^Department of Family and Community Medicine, University of Toronto, Toronto, ON, Canada; ^3^ICES, Toronto, ON, Canada; ^4^Department of Medicine, University of Toronto, Toronto, ON, Canada

**Keywords:** COVID-19 vaccination, major mental illness, substance use disorder, Ontario (Canada), disparities (health)

## Abstract

**Introduction:**

Patients with major mental illness (MMI) and substance use disorders (SUD) face barriers in accessing healthcare. In this population-based retrospective cohort study, we investigated the uptake of COVID-19 vaccination in Ontario, Canada among community-dwelling individuals receiving healthcare for major mental illness (MMI) and/or substance use disorders (SUD), comparing them to matched general population controls.

**Methods:**

Using linked health administrative data, we identified 337,290 individuals receiving healthcare for MMI and/or SUD as of 14 December 2020, matched by age, sex, and residential geography to controls without such healthcare. Follow-up extended until 31 December 2022 to document vaccination events.

**Results:**

Overall, individuals receiving healthcare for MMI and/or SUD (*N* = 337,290) had a slightly lower uptake of first (cumulative incidence 82.45% vs. 86.44%; hazard ratio [HR] 0.83 [95% CI 0.82–0.83]) and second dose (78.82% vs. 84.93%; HR 0.77 [95% CI 0.77–0.78]) compared to matched controls. Individuals receiving healthcare for MMI only (*n* = 146,399) had a similar uptake of first (87.96% vs. 87.59%; HR 0.97 [95% CI 0.96–0.98]) and second dose (86.09% vs. 86.05%, HR 0.94 [95% CI 0.93–0.95]). By contrast, individuals receiving healthcare for SUD only (*n* = 156,785) or MMI and SUD (*n* = 34,106) had significantly lower uptake of the first (SUD 78.14% vs. 85.74%; HR 0.73 [95% CI 0.72–0.73]; MMI & SUD 78.43% vs. 84.74%; HR 0.76 [95% CI 0.75–0.77]) and second doses (SUD 73.12% vs. 84.17%; HR 0.66 [95% CI 0.65–0.66]; MMI & SUD 73.48% vs. 82.93%; HR 0.68 [95% CI 0.67–0.69]).

**Discussion:**

These findings suggest that effective strategies to increase vaccination uptake for future COVID-19 and other emerging infectious diseases among community-dwelling people with SUD are needed.

## 1 Introduction

Access to healthcare facilities is a persistent challenge for patients with substance use disorders (SUD) and major mental illness (MMI), an umbrella term referring to specific mental health disorders (such as psychotic disorder, schizophrenia, or bipolar disorder), which interfere with daily living (1–3). Public health measures that reduced the transmission of SARS-CoV-2 during the COVID-19 pandemic had the unintended side effect of limiting supports and access to care for these individuals (4). Moreover, individuals with MMI and/or SUD also faced a higher burden of SARS-CoV-2 infection and adverse sequelae of infection compared to the general population (5–7). As a result, increasing the uptake of COVID-19 vaccination is key to reducing inequitable outcomes in this group.

In Ontario, Canada, COVID-19 vaccines were accessible from December 14, 2020, but for several months, the stock was very limited, forcing the provincial government to determine how to prioritize access. At the advice of Ontario's Vaccine Distribution Taskforce (8), Ontario decided to prioritize individuals based on risk factors for negative health outcomes following infection (for example, age and comorbidities) (8, 9), by likelihood of infection (for example, healthcare or essential worker status), and eventually by geography, as determined by early infection surveillance reports (8, 10, 11). People with MMI and/or SUD were included in Phase 2 of Ontario's vaccination strategy, becoming eligible for vaccination on the basis of having these conditions as of April 2021 (12). Vaccine administration, the responsibility of regional health units and municipalities, occurred primarily through specialized mass vaccination clinics, local pharmacies, and certain local primary care providers (13). In many areas, mobile clinics were also used to help vaccinate groups with anticipated barriers to access (13, 14). However, despite efforts to improve access through eligibility prioritization, other well-known barriers to uptake may have prevented individuals with MMI and/or SUD from benefiting with regards their eligibility prioritization. These barriers include low vaccine awareness and education (including tackling misinformation and pre-existing mistrust) (15, 16), lack of transportation to vaccination centers (15), inadequate internet access or technological literacy levels (17) needed to book appointments, or the numerous competing priorities with higher short-term urgency, such as securing basic needs such as shelter (18) and food (19).

In the present study, we assessed COVID-19 vaccination rates among community-dwelling individuals receiving healthcare facilities for MMI and/or SUD in Ontario as compared to matched controls, to better understand whether this population may have faced continued barriers to vaccine uptake.

## 2 Methods

### 2.1 Study design and setting

We conducted a retrospective population-based cohort study in Ontario, Canada (population = 14.2 million) (20), from 14 December 2020 to 31 December 2022. As in most parts of Canada, the majority of Ontarians (80%) reside in large urban centers, but there are also substantial populations in rural regions with varying levels of remote dwelling (21). In Ontario, COVID-19 vaccine products were obtained and supplied free of cost through the public healthcare system and received through mass vaccination clinics, pharmacies, and eventually through mobile vaccination units (13).

We used health administrative databases associated using unique encoded identifiers and analyzed at the ICES (formerly known as the Institute for Clinical Evaluative Sciences) (22). The ICES is a prescribed entity under Section 45 of Ontario's Personal Health Information Protection Act. This Act authorizes the ICES to collect personal health information, without consent, for the purpose of analysis or compiling statistical information with respect to the management of, evaluation or monitoring of, the allocation of resources to or planning for all or part of the healthcare system. In Ontario, healthcare is administered through a single-payer model, with universal coverage of medical services provided through the Ontario Health Insurance Plan (OHIP) (23). Thus, administrative databases for health services provided cover the vast majority (>99%) of the population. This study follows the Reporting of Studies Conducted Using Observational Routinely Collected Data (RECORD) reporting guidelines (see Supplementary material A) (24).

### 2.2 Data sources

We identified participants using a combination of the ICES Registered Persons Database, the Canadian Institute for Health Information Discharge Abstract Database, the National Ambulatory Care Reporting System, the Ontario Mental Health Reporting System, and the Ontario Health Insurance Plan claims database. We identified vaccination outcomes through the Ontario COVAXON database, which includes all vaccine doses administered in Ontario, as well as doses administered outside Ontario, for which the individual provided proof of vaccination and consent for inclusion into the database. Other covariates were drawn from a variety of databases, including the aforementioned sources, and also certain ICES-derived datasets that apply validated case definitions, such as the Ontario Asthma dataset (25); the Ontario Chronic Obstructive Pulmonary Disease cohort (26); the Ontario Diabetes dataset (27); the Ontario Hypertension dataset (28); the Ontario Congestive Heart Failure dataset (29); and the Ontario Dementia dataset (30). All data sources are further described in Supplementary material B.

### 2.3 Participants

We followed-up participants from 14 December 2020, the date of the first COVID-19 vaccination in Ontario (31), until 31 December 2022, the latest date for which complete data were available at the time of analysis. Potential participants in both groups were excluded if they were ineligible for OHIP coverage (for example, recent migrants to Ontario such as interprovincial migrants, refugees whose claims have not yet been accepted, or international migrants with short-term work permits, representing fewer than 1% of the population) (23), were not Ontario residents, or were potentially not in Ontario for the period of study (i.e., if they had no contact with the Ontario healthcare system within the past 5 years). Individuals were also excluded where geographic information on the postal code was unknown, as matching on geography was required to account for vaccine prioritization by area of residence (8, 10, 11) or where the individual resided in a long-term care facility within 120 days of start of follow-up, as such individuals had different prioritization and access to COVID-19 vaccination than community-dwelling individuals, our population of interest.

Participants comprised two groups. Our first group consisted of adults aged 18 to 105 who met the case definition of MMI and/or SUD within the 3 years prior to 14 December 2020. Individuals met the case definition for MMI if they were hospitalized in an acute or psychiatric inpatient facility or had at least three emergency department visits or physician service claims with a diagnosis of any psychotic disorder or bipolar disorder. We did not include depression in our definition of MMI due to the inability of administrative data to distinguish between depression and other conditions in outpatient data. Individuals met the case definition for SUD if they were hospitalized in acute or psychiatric inpatient facilities or had at least three emergency department visits or physician service claims with a diagnosis of substance use disorder. If individuals met case definitions for both MMI and SUD, they were flagged as having both conditions. Our case definitions were adapted from validated definitions for chronic psychotic illness (32). Full case definitions including eligible codes are available in Supplementary material C. Our second control group consisted of individuals alive as of 14 December 2020 without any healthcare contact for MMI or SUD in the past 3 years. Individuals with some eligible healthcare contact for MMI and/or SUD, but not enough to meet either case definition, were excluded from both study groups.

We matched potential participants without healthcare for MMI or SUD to participants with MMI and/or SUD at a 1:1 ratio, without replacement, by age (exact), sex-at-birth (exact), and geography (forward sortation area code or FSA, which are postal districts based on residential postal codes). We matched on age since age was a key eligibility criterion for vaccination prioritization, and we matched on sex-at-birth due to expected differences in prevalence as well as vaccination uptake (33). We matched on geography because vaccination was prioritized for certain forward sortation areas that were associated with excess infection transmission in Ontario (8, 10, 11). We did not match on comorbidities or other factors that significantly differed between groups in order to avoid potentially increasing the risk of including individuals with MMI and/or SUD who do not receive healthcare facilities for their condition(s) among the controls. Individuals were censored at entry into long-term care or death.

### 2.4 Outcomes

Our outcome of interest was time to receipt of any COVID-19 vaccine product, as recorded in the Ontario COVID-19 vaccine database (COVAXON). We considered time to first and second doses primarily, as in Ontario, the vast majority (>99%) of vaccine products were two-dose ones. Therefore, the receipt of two doses indicates a full primary course of vaccination.

### 2.5 Covariates

We obtained participants' demographic characteristics as of 14 December 2020, including age, sex-assigned-at-birth, rurality, and geography of the participant's residence (Local Health Integration Network), neighborhood-level income quintile, and neighborhood-level of racialized and newcomer populations. We further identified the presence of asthma (25), chronic obstructive pulmonary disorder (26), diabetes (27), congestive heart failure (28), hypertension (29), and dementia (30) using validated case definitions. Finally, we measured the participant's Charlson comorbidity index, used to predict mortality based on the presence and severity of comorbidities, by using a methodology (34) adapted for International Classification of Diseases, 10th revision (35) using patient hospitalizations from the past 5 years. The complete variable and validated case definitions are available in Supplementary material C.

### 2.6 Statistical analysis

We compared baseline characteristics between groups before and after matching. Due to the size of the cohorts, we used standardized differences (36), which assess differences between group means as a percentage of the pooled standard deviation to assess the significance of differences between groups. A difference of 10% or more was considered meaningful. We calculated the cumulative incidence of receiving first and second doses of COVID-19 vaccines by group membership, overall, and stratifying by type of exposure (MMI and/or SUD; MMI only; SUD only; or MMI and SUD). Finally, we used survival modeling to estimate the hazard ratio (HR) of receiving a first or second dose of COVID-19 vaccine in the MMI and/or SUD group over the observation period, compared to their matched control; again, these were repeated by the type of exposure. Models reported were Cox proportional hazards models rather than subdistributional hazard models, taking into account the competing risk of death, as the latter did not meaningfully change results.

We ran a number of sensitivity analyses to ensure robustness of results. First, we ran adjusted survival models adjusting for a neighborhood level income, Charlson Comorbidity Index, and both neighborhood level income and Charlson Comorbidity Index. Second, we repeated our entire analysis with a more sensitive case definition (requiring only one eligible hospitalization, ED or outpatient visit to be considered part of the MMI and/or SUD cohort). Results of sensitivity analyses are presented in the Supplementary material.

In all outputs, small cells (less than or equal to five) were suppressed to protect patient privacy. All statistical tests were two-tailed with p < 0.05 set as the level of significance; analyses were conducted using SAS Enterprise Guide v 7.1.

### 2.7 Ethical review

This study received ethics approval from the Health Sciences Research Ethics Board at the University of Toronto (RIS Protocol # 41528).

## 3 Results

**Figure 1:**
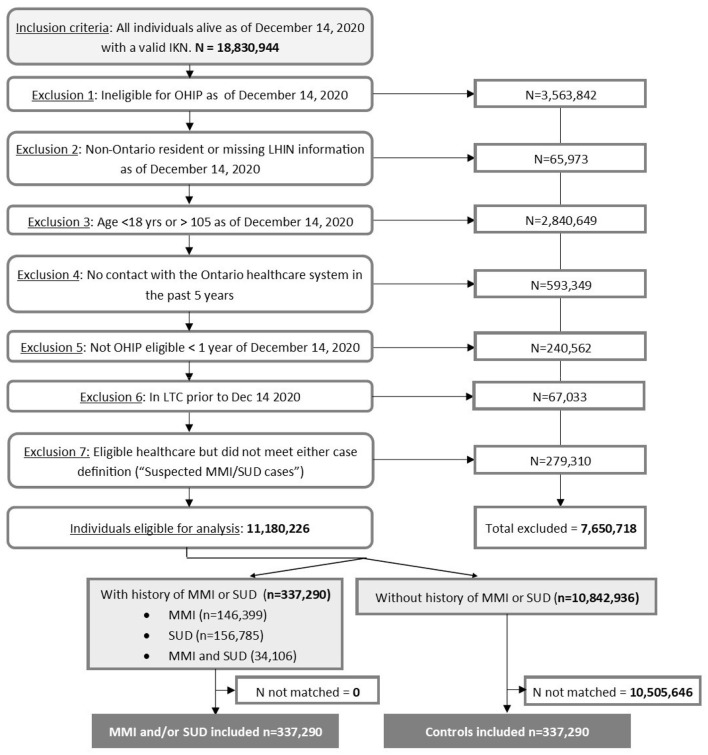
Cohort build inclusion and exclusion criteria.

We identified 337,290 eligible community-dwelling people receiving healthcare facilities for MMI and/or SUD and 10,842,936 eligible community-dwelling people without healthcare facilities for MMI or SUD (Figure 1). Supplementary material D, Table 1 describes the unmatched cohorts. Briefly, individuals receiving healthcare facilities for MMI and/or SUD were younger, more likely to be male, reside in neighborhoods with the lowest income quintile, and have substantially higher rates of asthma, COPD, and dementia compared to controls. They were also more likely to have a higher Charlson score. After matching, we included 337,290 community-dwelling individuals in each group (Figure 1). Of these, a total of 146,399 participants had MMI only; 156,785 participants had SUD only; and 34,106 participants had both MMI and SUD.

**Table 1:** Matched baseline characteristics, by group membership.

	**Total (*n* = 674,580)**	**MMI/SUD Patients (*n* = 337,290)**	**Controls (*n* = 337,290)**	**% SD**
**Age**				
Mean (SD)	46.08 (16.59)	46.08 (16.59)	46.08 (16.59)	0
Median (IQR)	44 (32–58)	44 (32–58)	44 (32–58)	0
**Age category**, ***N*** **(%)**				
18–29 years	124,627 (18.5%)	62,295 (18.5%)	62,332 (18.5%)	0
30–39 years	148,162 (22.0%)	74,093 (22.0%)	74,069 (22.0%)	0
40–49 years	124,044 (18.4%)	62,022 (18.4%)	62,022 (18.4%)	0
50–59 years	125,449 (18.6%)	62,717 (18.6%)	62,732 (18.6%)	0
60–69 years	90,833 (13.5%)	45,436 (13.5%)	45,397 (13.5%)	0
70–79 years	41,138 (6.1%)	20,562 (6.1%)	20,576 (6.1%)	0
80+ years	20,327 (3.0%)	10,165 (3.0%)	10,162 (3.0%)	0
**Sex**, ***N*** **(%)**				
Female	301,098 (44.6%)	150,549 (44.6%)	150,549 (44.6%)	0
Male	373,482 (55.4%)	186,741 (55.4%)	186,741 (55.4%)	0
**Rurality**, ***N*** **(%)**				
Urban	599,973 (88.9%)	299,149 (88.7%)	300,824 (89.2%)	2.0
Rural	71,494 (10.6%)	35,893 (10.6%)	35,601 (10.6%)	0.3
Missing	3,113 (0.5%)	2,248 (0.7%)	865 (0.3%)	6.1
**LHIN**, ***N*** **(%)**				
Erie St. Clair	40,525 (6.0%)	20,271 (6.0%)	20,254 (6.0%)	0
Southwest	49,243 (7.3%)	24,598 (7.3%)	24,645 (7.3%)	0.1
Waterloo Wellington	34,902 (5.2%)	17,474 (5.2%)	17,428 (5.2%)	0.1
Hamilton Niagara Haldimand Brant	79,750 (11.8%)	39,874 (11.8%)	39,876 (11.8%)	0
Central West	35,341 (5.2%)	17,718 (5.3%)	17,623 (5.2%)	0.1
Mississauga Halton	43,981 (6.5%)	21,994 (6.5%)	21,987 (6.5%)	0
Toronto Central	78,404 (11.6%)	39,255 (11.6%)	39,149 (11.6%)	0.1
Central	60,015 (8.9%)	29,952 (8.9%)	30,063 (8.9%)	0.1
Central East	70,059 (10.4%)	34,927 (10.4%)	35,132 (10.4%)	0.2
South East	28,292 (4.2%)	14,245 (4.2%)	14,047 (4.2%)	0.3
Champlain	61,374 (9.1%)	30,656 (9.1%)	30,718 (9.1%)	0.1
North Simcoe Muskoka	27,460 (4.1%)	13,711 (4.1%)	13,749 (4.1%)	0.1
North East	42,327 (6.3%)	21,137 (6.3%)	21,190 (6.3%)	0.1
North West	22,907 (3.4%)	11,478 (3.4%)	11,429 (3.4%)	0.1
**Neighborhood-level income quintile, N (%)**				
Quintile 1	188,505 (27.9%)	108,118 (32.1%)	80,387 (23.8%)	18
Quintile 2	145,147 (21.5%)	73,346 (21.7%)	71,801 (21.3%)	1.1
Quintile 3	124,285 (18.4%)	59,168 (17.5%)	65,117 (19.3%)	5.0
Quintile 4	108,019 (16.0%)	48,933 (14.5%)	59,086 (17.5%)	8.0
Quintile 5	105,206 (15.6%)	45,310 (13.4%)	59,896 (17.8%)	12
Missing	3,418 (0.5%)	2,415 (0.7%)	1,003 (0.3%)	6.0
**Ontario Marginalization Index*****—*****Newcomers and racialized populations**, ***N*** **(%)**				
Quintile 1	116,406 (17.3%)	56,938 (16.9%)	59,468 (17.6%)	2.0
Quintile 2	123,472 (18.3%)	60,539 (17.9%)	62,933 (18.7%)	2.0
Quintile 3	132,467 (19.6%)	65,944 (19.6%)	66,523 (19.7%)	0.4
Quintile 4	139,181 (20.6%)	69,883 (20.7%)	69,298 (20.5%)	0.4
Quintile 5	149,300 (22.1%)	75,067 (22.3%)	74,233 (22.0%)	0.6
Missing	13,754 (2.0%)	8,919 (2.6%)	4,835 (1.4%)	9.0
Asthma, *N* (%)	132,429 (19.6%)	78,213 (23.2%)	54,216 (16.1%)	18
CHF, *N* (%)	17,789 (2.6%)	12,369 (3.7%)	5,420 (1.6%)	13
COPD, *N* (%)	26,852 (4.0%)	20,560 (6.1%)	6,292 (1.9%)	22
Hypertension, *N* (%)	162,954 (24.2%)	89,582 (26.6%)	73,372 (21.8%)	11
Diabetes, *N* (%)	84,115 (12.5%)	49,111 (14.6%)	35,004 (10.4%)	13
Dementia, *N* (%)	14,610 (2.2%)	12,285 (3.6%)	2,325 (0.7%)	20
**Charlson comorbidity index**, ***N*** **(%)**				
0/no hosp	594,535 (88.1%)	279,937 (83.0%)	314,598 (93.3%)	32
1	36,938 (5.5%)	26,586 (7.9%)	10,352 (3.1%)	21
2	20,671 (3.1%)	13,642 (4.0%)	7,029 (2.1%)	11
3+	22,436 (3.3%)	17,125 (5.1%)	5,311 (1.6%)	20

MMI, major mental illness; SUD, substance use disorder; SD, standardized difference; CHF, congestive heart failure; COPD, chronic obstructive pulmonary disease.

Characteristics of matched participants are provided in Table 1. After matching, few demographic characteristics between groups remained significantly different; however, individuals receiving healthcare facilities for MMI and/or SUD remained significantly more likely to reside in neighborhoods with the lowest income quintile (32.1% vs. 23.8%). They were also more likely to have a higher Charlson Comorbidity Index, as well as asthma (23.2% vs. 16.1%), COPD (6.1% vs. 1.9%), congestive heart failure (3.7% vs. 1.6%), diabetes (14.6% vs. 10.4%), hypertension (26.6% vs. 21.8%), and dementia (3.6% vs. 0.7%).

**Figure 2:**
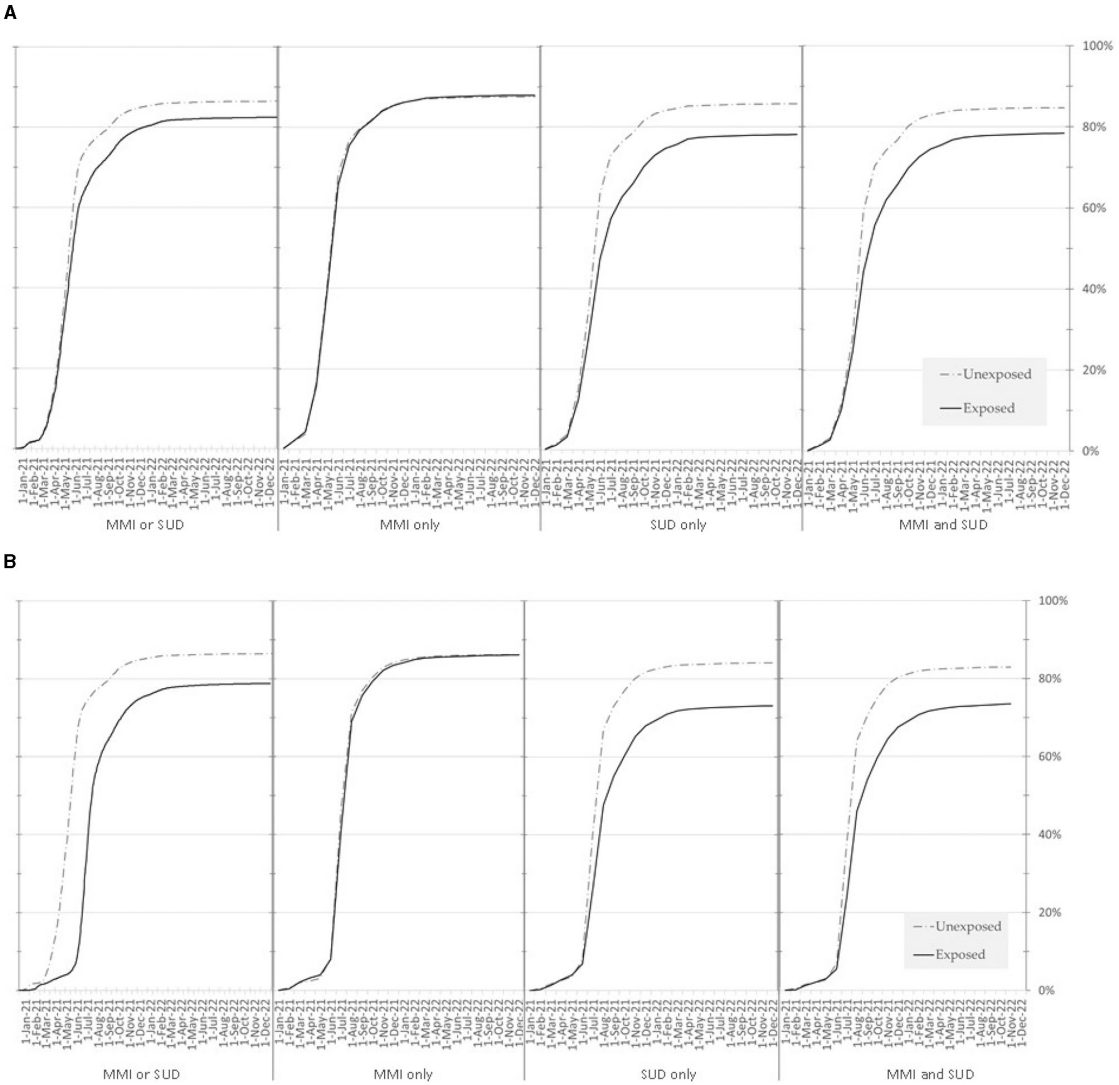
**(A)** Cumulative incidence of the first COVID-19 vaccine dose among patients with MMI and/or SUD and matched controls, between 14 December 2020 and 31 December 2022. MMI, major mental illness; SUD, substance use disorder. **(B)** Cumulative incidence of the second COVID-19 vaccine dose among patients with MMI and/or SUD and matched controls, between 14 December 2020 and 31 December 2022. MMI, major mental illness; SUD, substance use disorder.

Figures 2A, B show the cumulative incidence of receiving a first and second dose of vaccine by exposure status and subgroup. By 31 December 2022, individuals with MMI and/or SUD had a cumulative incidence of first and second dose of 82.45% and 78.82%, respectively, compared to 86.44% and 84.93% among controls, respectively. However, much of the difference appears to be driven by the uptake among individuals with SUD (with or without MMI). Individuals with only MMI had a cumulative incidence of first and second dose of 87.96% and 86.09%, respectively, very similar to 87.59% and 86.05% among controls. By contrast, individuals with only SUD had a cumulative incidence of first and second dose of 78.14% and 73.12%, respectively, compared to 85.74% and 84.17% among controls, and individuals with both MMI & SUD had a cumulative incidence of cumulative incidence of first and second dose of 78.43% and 73.48%, respectively, compared to 84.74% and 82.93% among controls. In both SUD subgroups, uptake for both first and second doses began to diverge when uptake was approximately 20%, in May (first dose) and July (second dose) 2021.

Table 2 shows the results of Cox proportional hazard models assessing the association between group membership and vaccine uptake, by dose and subgroup. All subgroups had statistically significant lower first- and second-dose uptake compared to their matched controls, but the difference between MMI patients and their controls was relatively small (first dose HR 0.971 [95% CI 0.964–0.979]; second dose HR 0.940 [95% CI 0.933–0.947]); while a larger difference was observed in patients with SUD only (first dose HR 0.728 [95% CI 0.723–0.734]; second dose HR 0.657 [95% CI 0.652–0.662]) or patients with both MMI and SUD (first dose HR 0.758 [95% CI 0.746–0.770]; second dose HR 0.682 [95% CI 0.671–0.694]), compared to their corresponding controls.

**Table 2:** Unadjusted Cox proportional hazards model assessing the association between major mental illness and/or substance use disorder and receipt of a first or second dose of any COVID-19 vaccine before the end of the observation period (31 December 2022).

**Group**	**Dose #**	**Unadjusted HR^a^**	**95% CI**	***P*-value**
Major mental illness or substance use disorder	1st	0.828	0.824–0.832	< 0.0001
	2nd	0.771	0.767–0.775	< 0.0001
Major mental illness only	1st	0.971	0.964–0.979	< 0.0001
	2nd	0.940	0.933–0.947	< 0.0001
Substance use disorder only	1st	0.728	0.723–0.734	< 0.0001
	2nd	0.657	0.652–0.662	< 0.0001
Major mental illness and substance use disorder	1st	0.758	0.746–0.770	< 0.0001
	2nd	0.682	0.671–0.694	< 0.0001

^a^Reference group = controls matched on age, sex, and geography.

Supplementary Table 2 presents a sensitivity analysis adjusting the MMI/SUD models for neighborhood income, Charlson Comorbidity Index, and both neighborhood income and Charlson Comorbidity Index, showing a relatively less impact of these variables on first or second dose vaccine uptake. Supplementary Table 3, Supplementary Figures 1, 2 present results when using a case definition of MMI and/or SUD that required only one eligible healthcare encounter. This less stringent case definition approximately increased the cohort size by twofold; otherwise, the cumulative incidence figures (Supplementary Figure 1, 2) and model outputs (Supplementary Table 3) were similar to those presented in the main analysis.

## 4 Discussion

We found that community-dwelling individuals receiving healthcare facilities for major mental illness had only slightly lower COVID-19 vaccination uptake as compared to matched controls, but that community-dwelling individuals receiving healthcare facilities for either substance use disorder or combined MMI and SUD were less likely to be vaccinated by the study end date. Results were robust, irrespective of the case definition used or by adjustment for neighborhood income or level of comorbidity. Through most of the follow-up [April 2021 onward, when Phase 2 of Ontario's vaccination plan began (12)], individuals with any mental health diagnosis or substance use disorder were eligible to receive COVID-19 vaccination on a priority basis in Ontario (9, 12). Furthermore, as this group had substantially higher comorbidity rates than matched controls, many would have been also prioritized for vaccination due to physical comorbidity risk factors, which were also prioritized in Ontario's vaccination strategy (9). Despite this, vaccination rates for the SUD and MMI & SUD groups lagged substantially behind that of matched controls, with uptake for both doses diverging once groups had a cumulative incidence of about 20%.

Previous reports concur with our findings, showing a lack of disparity in COVID-19 vaccination among people with MMI and disparities for people with SUD. People with major mental illness in other studies had vaccination rates ranging from only slightly lower (37), approximately similar (38, 39), or somewhat higher (40) than comparators in numerous settings. By contrast, most studies about individuals who use drugs report substantial COVID-19 vaccine hesitancy (41–43) and lower vaccine uptake than direct or indirect comparisons with general population comparators (44–48).

Despite both groups experiencing substantial stigma, perceptions of individuals with mental illness have changed substantially in the past decades, as understandings of the medical underpinnings of their condition have evolved (49). Moreover, individuals with substance use disorder continue to be frequently viewed through a moralistic lens, with their condition seen as a result of personal choice or moral failing rather than the complex interplay of genetic, environmental, and psychological factors (49). As a result, health professionals have been found to often hold stigmatizing views toward people with substance use disorders, which can affect the quality of care provided and feelings of patients toward healthcare (49, 50). This provides an important context to the literature about COVID-19 vaccine hesitancy and barriers to uptake, specific to people who use drugs. A recent national study (51) in Canada suggests that barriers to COVID-19 vaccination among people who use drugs include a lack of knowledge about the benefits and risks associated with the vaccine but also significant distrust toward government and healthcare agencies, and skepticism about the effectiveness of a vaccine so rapidly developed. Crucially, in this study and in other related work, people who use drugs trust information most when it is shared by peers (52), community leaders (53, 54), individuals with lived experience, harm reduction workers (55), and trusted healthcare providers (51), rather than by government or health authorities.

By contrast, Ontario took a top–down approach to its COVID-19 vaccination strategy, with the provincial government and local public health authorities organizing most of the official communication pertaining to vaccination. This was, unfortunately, judged to be disorganized and inconsistent by the Office of the Auditor General of Ontario, leading to public confusion and misunderstandings about the risks and benefits of COVID-19 vaccination (56). In combination with widespread misinformation around COVID-19 vaccine products and substantial distrust of government or healthcare officials generally, confidence in vaccination among individuals with substance use disorder was likely undermined (56). Additionally, although Public Health Ontario (a provincial agency) recommended communities adopt an individualized, culturally safe and trauma-informed approach to COVID-19 vaccination (57) and the province eventually promoted targeted strategies emphasizing accessibility (14), vaccination administration was organized by local public health units, (13) with the result that each local area in practice engaged with marginalized communities only to the extent possible by local resources and planning. Toronto, for example, invested significantly to enhance outreach through their Vaccine Engagement Team (VET) (58), which resulted in excellent local vaccination rates (59) as well as significantly reduced gaps in vaccination among groups with higher rates of substance use disorder, such as homeless people (60). Such approaches have also been used elsewhere with success (52–54), but were not very common across Canada (51, 56).

### 4.1 Strengths and limitations

Our study benefits from leveraging linked health administrative data which follow the vast majority of the Ontario population (that is, those eligible for OHIP coverage, representing over 99% of Ontario residents), giving us a quasi-population-level assessment of vaccination uptake among community-dwelling people receiving healthcare facilities for MMI and/or SUD. However, OHIP eligibility does not include recent migrants to Ontario such as interprovincial migrants, refugees whose claims have not yet been accepted, or international migrants with short-term work permits (23). The prevalence of major mental illness and substance use disorder may differ in some of these groups compared to the general population (61); therefore, results should only be extended to Ontarians with OHIP coverage.

Furthermore, our case definition of major mental illness and substance use disorder relies on the interaction with the healthcare system. Previous work has shown that a significant number of individuals with major mental illness or who use drugs experience stigma during healthcare interactions (62), which can result in healthcare avoidance; healthcare-avoidant individuals with MMI and/or SUD might be more likely to be distrustful of healthcare deemed non-essential like COVID-19 vaccination. Thus, our results may understate the true disparity in vaccine uptake in these groups. Combined with a lack of validation work to verify the case definition performance of our MMI and SUD constructs, our results should only be generalized to community-dwelling people with MMI and/or SUD who use healthcare.

Finally, our definition of MMI did not include codes for major depressive disorder, largely due to the inability of outpatient administrative databases in cleanly separating out this condition from other conditions not part of MMI (such as anxiety). As a result, findings may not be representative of results for individuals with major depressive disorder.

## 5 Conclusions

Community-dwelling people in Ontario receiving healthcare for SUD or combined MMI and SUD have lower COVID-19 vaccine uptake than controls matched by age, sex-at-birth, and geography. Our results suggest that efforts to improve vaccination uptake need to target the specific concerns and barriers faced by people who use drugs. Future work should determine the specific concerns and barriers to vaccination faced by people who use drugs in Ontario and assess the impact of strategies targeting such barriers and concerns, such as community-led outreach by trusted individuals.

## Data availability statement

The datasets presented in this article are not readily available because the dataset from this study is held securely in coded form at ICES. While legal data sharing agreements between ICES and data providers (e.g., healthcare organizations and government) prohibit ICES from making the dataset publicly available, access may be granted to those who meet pre-specified criteria for confidential access, available at www.ices.on.ca/DAS (email: das@ices.on.ca). The full dataset creation plan and underlying analytic code are available from the authors upon request, understanding that the computer programs may rely upon coding templates or macros that are unique to ICES and are therefore either inaccessible or may require modification. Requests to access data, the dataset creation plan or underlying analytic code may be sent to the corresponding author. Requests to access the datasets should be directed to das@ices.on.ca.

## Ethics statement

The studies involving humans were approved by Health Sciences Research Ethics Board at the University of Toronto. The studies were conducted in accordance with the local legislation and institutional requirements. Written informed consent for participation was not required from the participants or the participants' legal guardians/next of kin in accordance with the national legislation and institutional requirements.

## Author contributions

LR: Validation, Visualization, Writing – original draft, Writing – review & editing. AH: Conceptualization, Methodology, Writing – review & editing. VA: Data curation, Formal analysis, Methodology, Software, Validation, Writing – review & editing. MC: Methodology, Project administration, Validation, Writing – review & editing. SH: Conceptualization, Methodology, Supervision, Validation, Writing – review & editing.
