# Supplementary material for: Uptake of COVID-19 vaccination among community-dwelling individuals receiving healthcare for substance use disorder and major mental illness: a matched retrospective cohort study
Source: Front Public Health. 2024 Jul 5;12:1426152. doi: 10.3389/fpubh.2024.1426152 (PMC11257932; doi:10.3389/fpubh.2024.1426152)
Supplement: Supplementary file 1 [file Data_Sheet_1.docx]

**Supplement A: The RECORD statement – checklist of items, extended from the STROBE statement, that should be reported in observational studies using routinely collected health data.**

|  | **Item No.** | **STROBE items** | **Location in manuscript where items are reported** | **RECORD items** | **Location in manuscript where items are reported** |
| --- | --- | --- | --- | --- | --- |
| **Title and abstract** | | | | | |
|  | 1 | (a) Indicate the study’s design with a commonly used term in the title or the abstract (b) Provide in the abstract an informative and balanced summary of what was done and what was found | Title | RECORD 1.1: The type of data used should be specified in the title or abstract. When possible, the name of the databases used should be included.  RECORD 1.2: If applicable, the geographic region and timeframe within which the study took place should be reported in the title or abstract.  RECORD 1.3: If linkage between databases was conducted for the study, this should be clearly stated in the title or abstract. | 1.1, 1.2, 1.3 : Abstract |
| **Introduction** | | | | | |
| Background rationale | 2 | Explain the scientific background and rationale for the investigation being reported | Background |  |  |
| Objectives | 3 | State specific objectives, including any prespecified hypotheses | Background |  |  |
| **Methods** | | | | | |
| Study Design | 4 | Present key elements of study design early in the paper | Methods: Study Design and Setting |  |  |
| Setting | 5 | Describe the setting, locations, and relevant dates, including periods of recruitment, exposure, follow-up, and data collection | Methods: Study Design and Setting |  |  |
| Participants | 6 | *(a) Cohort study* - Give the eligibility criteria, and the sources and methods of selection of participants. Describe methods of follow-up  *Case-control study* - Give the eligibility criteria, and the sources and methods of case ascertainment and control selection. Give the rationale for the choice of cases and controls  *Cross-sectional study* - Give the eligibility criteria, and the sources and methods of selection of participants  *(b) Cohort study* - For matched studies, give matching criteria and number of exposed and unexposed  *Case-control study* - For matched studies, give matching criteria and the number of controls per case | Methods: Participants and Data sources | RECORD 6.1: The methods of study population selection (such as codes or algorithms used to identify subjects) should be listed in detail. If this is not possible, an explanation should be provided.  RECORD 6.2: Any validation studies of the codes or algorithms used to select the population should be referenced. If validation was conducted for this study and not published elsewhere, detailed methods and results should be provided.  RECORD 6.3: If the study involved linkage of databases, consider use of a flow diagram or other graphical display to demonstrate the data linkage process, including the number of individuals with linked data at each stage. | Methods: Participants and Data sources |
| Variables | 7 | Clearly define all outcomes, exposures, predictors, potential confounders, and effect modifiers. Give diagnostic criteria, if applicable. | Methods: Population, Covariates, and  Outcomes | RECORD 7.1: A complete list of codes and algorithms used to classify exposures, outcomes, confounders, and effect modifiers should be provided. If these cannot be reported, an explanation should be provided. | Methods: Population, Covariates, and  Outcomes;  Supplement |
| Data sources/ measurement | 8 | For each variable of interest, give sources of data and details of methods of assessment (measurement).  Describe comparability of assessment methods if there is more than one group | N/A |  |  |
| Bias | 9 | Describe any efforts to address potential sources of bias | N/A |  |  |
| Study size | 10 | Explain how the study size was arrived at | N/A |  |  |
| Quantitative variables | 11 | Explain how quantitative variables were handled in the analyses. If applicable, describe which groupings were chosen, and why | N/A |  |  |
| Statistical methods | 12 | (a) Describe all statistical methods, including those used to control for confounding  (b) Describe any methods used to examine subgroups and interactions  (c) Explain how missing data were addressed  (d) *Cohort study* - If applicable, explain how loss to follow-up was addressed  *Case-control study* - If applicable, explain how matching of cases and controls was addressed  *Cross-sectional study* - If applicable, describe analytical methods taking account of sampling strategy  (e) Describe any sensitivity analyses | Methods, Statistical Analysis |  |  |
| Data access and cleaning methods |  | .. |  | RECORD 12.1: Authors should describe the extent to which the investigators had access to the database population used to create the study population.  RECORD 12.2: Authors should provide information on the data cleaning methods used in the study. | 12.1 N/A  12.2 N/A |
| Linkage |  | .. |  | RECORD 12.3: State whether the study included person-level, institutional-level, or other data linkage across two or more databases. The methods of linkage and methods of linkage quality evaluation should be provided. | Methods: Study Design and Setting |
| **Results** | | | | | |
| Participants | 13 | (a) Report the numbers of individuals at each stage of the study (*e.g.*, numbers potentially eligible, examined for eligibility, confirmed eligible, included in the study, completing follow-up, and analysed)  (b) Give reasons for non-participation at each stage.  (c) Consider use of a flow diagram | Results, Figure 1 | RECORD 13.1: Describe in detail the selection of the persons included in the study (*i.e.,* study population selection) including filtering based on data quality, data availability and linkage. The selection of included persons can be described in the text and/or by means of the study flow diagram. | N/A |
| Descriptive data | 14 | (a) Give characteristics of study participants (*e.g.*, demographic, clinical, social) and information on exposures and potential confounders  (b) Indicate the number of participants with missing data for each variable of interest  (c) *Cohort study* - summarise follow-up time (*e.g.*, average and total amount) | Results |  |  |
| Outcome data | 15 | *Cohort study* - Report numbers of outcome events or summary measures over time  *Case-control study* - Report numbers in each exposure category, or summary measures of exposure  *Cross-sectional study* - Report numbers of outcome events or summary measures | Results |  |  |
| Main results | 16 | (a) Give unadjusted estimates and, if applicable, confounder-adjusted estimates and their precision (e.g., 95% confidence interval). Make clear which confounders were adjusted for and why they were included  (b) Report category boundaries when continuous variables were categorized  (c) If relevant, consider translating estimates of relative risk into absolute risk for a meaningful time period | Results |  |  |
| Other analyses | 17 | Report other analyses done—e.g., analyses of subgroups and interactions, and sensitivity analyses | N/A |  |  |
| **Discussion** | | | | | |
| Key results | 18 | Summarise key results with reference to study objectives | Discussion par 1 |  |  |
| Limitations | 19 | Discuss limitations of the study, taking into account sources of potential bias or imprecision. Discuss both direction and magnitude of any potential bias | Discussion: Limitations | RECORD 19.1: Discuss the implications of using data that were not created or collected to answer the specific research question(s). Include discussion of misclassification bias, unmeasured confounding, missing data, and changing eligibility over time, as they pertain to the study being reported. | Discussion: Limitations |
| Interpretation | 20 | Give a cautious overall interpretation of results considering objectives, limitations, multiplicity of analyses, results from similar studies, and other relevant evidence | Discussion par 2, 3 |  |  |
| Generalisability | 21 | Discuss the generalisability (external validity) of the study results | Discussion par 2, 3 |  |  |
| **Other Information** | | | | | |
| Funding | 22 | Give the source of funding and the role of the funders for the present study and, if applicable, for the original study on which the present article is based | Funding statements |  |  |
| Accessibility of protocol, raw data, and programming code |  | .. |  | RECORD 22.1: Authors should provide information on how to access any supplemental information such as the study protocol, raw data, or programming code. | Data availability statement |

*Reference: Benchimol EI, Smeeth L, Guttmann A, Harron K, Moher D, Petersen I, Sørensen HT, von Elm E, Langan SM, the RECORD Working Committee. The REporting of studies Conducted using Observational Routinely-collected health Data (RECORD) Statement. *PLoS Medicine* 2015; in press.

*Checklist is protected under Creative Commons Attribution ([CC BY](http://creativecommons.org/licenses/by/4.0/)) license.

**Supplement B: Description of data sources**

| Data source | Description |
| --- | --- |
| **ICES Registered Persons Database (RPDB)** | The RPDB database provides demographic information on any individual who has ever received an Ontario health card number, including date of birth and death (if applicable), sex-at-birth, and postal code for each year. |
| **CIHI Discharge Abstract Database (DAD)** | DAD captures administrative (institution-hospital number, admission category, length of stay, disposition), clinical (diagnoses, procedures, physician) and demographic information (patient gender, date of birth, postal code, county and residence code), on hospital discharges including deaths, sign-outs and transfers. |
| **National Ambulatory Care Reporting System (NACRS)** | NACRS captures information on patient visits to hospital and community based ambulatory care such as day surgery, outpatient clinics and emergency departments within Ontario. |
| **Ontario Mental Health Reporting System (OMHRS)** | OHMRS collects data on patients in adult designated inpatient mental health beds. |
| **Ontario Health Insurance Plan (OHIP) claims** | The OHIP claims database contains most claims paid for by the Ontario Health Insurance Plan. The data covers all health care providers who can claim under OHIP (this includes physicians, groups, laboratories, and out-of-province providers) for the purposes of maintaining a record of the patient and physician, services provided, date of the service, associated diagnosis, and fees paid. Excludes services provided by Community Health Centres. |
| **Ontario COVID-19 Vaccine Database (COVAXON)** | The Ontario COVID-19 vaccine database contains information on all COVID-19 vaccination events for the purpose of monitoring vaccination uptake. |
| **Ontario Asthma Database (ASTHMA)** | The Ontario Asthma Database is contains all Ontario asthma patients identified through a validated case definition^2^ since 1991 through primary care patient records and hospital administrative data. |
| **Chronic Obstructive Pulmonary Disease Database (COPD)** | The Ontario Chronic Obstructive Pulmonary Disease Database contains all Ontario COPD patients identified through a validated case definition^3^ since 1991 through primary care patient records and hospital administrative data |
| **Ontario Diabetes Database (ODD)** | The Ontario Diabetes Database contains all Ontario diabetes patients identified through a validated case definition^4^ since 1991 through primary care patient records and hospital administrative data. |
| **Congestive Heart Failure Database (CHF)** | The Ontario Congestive Heart Failure Database contains all Ontario individuals with CHF identified through a validated case definition^5^ since 1991 through primary care patient records and hospital administrative data. |
| **Ontario Hypertension Database (HYPER)** | The Ontario Hypertension Database contains all Ontario individuals with hypertension identified through a validated case definition^6^ since 1991 through primary care patient records and hospital administrative data |
| **Ontario Dementia database (DEMENTIA)** | The Ontario Dementia Database contains all Ontario individuals with dementia identified through a validated case definition^8^ since 1991. |
|  |  |

**Supplement C – Full variable definitions**

| **Variable** | Definition |
| --- | --- |
| **Major mental illness** | One hospitalization or three outpatient/ED visits in the past three years for psychotic disorder or bipolar disorder. Eligible codes include F06.0-2, F20.x, F22.x-F29.x, F30.x, F31.x, F34.0, or F53.1 (ICD-10); 295, 297, 298 or 296 (OHIP diagnostic codes); Q020 or Q021 (OHIP billing codes); or 293.81, 293.82, 295.x, 296.0x, 296.1x, 296.4x, 296.5x, 296.7x, 296.8x, 297.x, 298.x, 301.13 (DSM-V). |
| **Substance use disorder** | One hospitalization or three outpatient/ED visits in the past three years for substance use disorder. Eligible codes include F10.x-F19.x, F55.x (ICD-10); 291, 292, 303, 304 (OHIP diagnostic codes); K680, A957 (OHIP billing codes); or 291.x, 292.x, 303.x, 304.x, 305.x (DSM-V) |
| **Age** | Participant’s age as calculated from the ICES Registered Persons Database date of birth and cohort entry date. Expressed numerically or as age groups (‘16 to 29 years’; ‘30 to 49 years’; ‘50 to 69 years’; and ‘70+ years’). |
| **Sex-at-birth** | Participant’s sex-at-birth as recorded in the ICES Registered Persons Database. Expressed as ‘Male’ or ‘Female’. |
| **Geography: Local Health Integration Network** | Participant’s residence Local Health Integration Network (LHIN), the health authorities responsible for regional administration of public healthcare services in Ontario. |
| **Neighbourhood-level income quintile** | Neighbourhood-level income quintile are assigned based on the postal code of the patient’s residence at index. Using Statistics Canada's Postal Code Conversion File Plus (PCCF+), postal codes are linked to census geography at the dissemination area level (the smallest area at which population characteristics from the Canadian Census are reported). Each dissemination area was assigned to an nbhd-level income quintile according to the 2016 census household income of the residents of that area and compared against incomes of other dissemination areas in the region. |
| **Neighbourhood-level % of racialized and newcomer populations** | Neighbourhood-level % of racialized and newcomer populations is an Ontario Marginalization Index measure that estimates the proportion of newcomers and/or nonwhite, non-Indigenous populations in a region, and relates to the potential impacts of racialization and xenophobia on residents. This quintile is assigned based on the postal code of the patient’s residence at index. Using Statistics Canada's Postal Code Conversion File Plus (PCCF+), postal codes are linked to census geography at the dissemination area level (the smallest area at which population characteristics from the Canadian Census are reported). Each dissemination area was assigned to a neighbourhood-level % of racialized and newcomer populations according to the 2016 census of the residents of that area and compared against other dissemination areas in the region.  Please note that racialized and newcomer populations are grouped together in this variable, and cannot measure differences between these two populations. |
| **Asthma** | Presence in the ICES Asthma Database at any point prior to cohort entry |
| **Chronic lung disease** | Presence in the ICES COPD Database at any point prior to cohort entry. |
| **Diabetes** | Presence in the ICES Diabetes Database at any point prior to cohort entry |
| **Chronic heart disease** | Presence in the ICES CHF Database at any point prior to cohort entry |
| **Hypertension** | Presence in the ICES Hypertension Database at any point prior to cohort entry. |
| **Dementia** | Presence in the ICES Dementia Database at any point prior to cohort entry |
| **Charlson comorbidity index category** | Participant’s Charlson comorbidity index, categorized into ‘Zero/No hospitalizations’, ‘1’, ‘2’ or ‘3+’, using hospitalization data from the past five years. |

**Supplement D: Supplementary tables**

**Supplement Table 1 – Unmatched baseline characteristics, by group membership**

|  | **Total (n=11,180,226)** | **MMI/SUD Patients (n=337,290)** | **Unexposed Controls (n=10,842,936)** | **SD** |
| --- | --- | --- | --- | --- |
| Age |  |  |  |  |
| Mean (SD) | 49.02 (18.41) | 46.08 (16.59) | 49.11 (18.46) | 0.17 |
| Median (IQR) | 49 (33-63) | 44 (32-58) | 49 (33-63) | 0.17 |
| Age category, N (%) |  |  |  |  |
| 18-29 years | 2,032,567 (18.2%) | 62,295 (18.5%) | 1,970,272 (18.2%) | 0.01 |
| 30-39 years | 1,916,330 (17.1%) | 74,093 (22.0%) | 1,842,237 (17.0%) | 0.13 |
| 40-49 years | 1,788,811 (16.0%) | 62,022 (18.4%) | 1,726,789 (15.9%) | 0.07 |
| 50-59 years | 1,984,597 (17.8%) | 62,717 (18.6%) | 1,921,880 (17.7%) | 0.02 |
| 60-69 years | 1,738,559 (15.6%) | 45,436 (13.5%) | 1,693,123 (15.6%) | 0.06 |
| 70-79 years | 1,119,350 (10.0%) | 20,562 (6.1%) | 1,098,788 (10.1%) | 0.15 |
| 80+ years | 600,012 (5.4%) | 10,165 (3.0%) | 589,847 (5.4%) | 0.12 |
| Sex, N (%) |  |  |  |  |
| Female | 5,742,903 (51.4%) | 150,549 (44.6%) | 5,592,354 (51.6%) | 0.14 |
| Male | 5,437,323 (48.6%) | 186,741 (55.4%) | 5,250,582 (48.4%) | 0.14 |
| Rurality, N (%) |  |  |  |  |
| Urban | 10,001,178 (89.5%) | 299,149 (88.7%) | 9,702,029 (89.5%) | 0.03 |
| Rural | 1,152,510 (10.3%) | 35,893 (10.6%) | 1,116,617 (10.3%) | 0.01 |
| Missing | 26,538 (0.2%) | 2,248 (0.7%) | 24,290 (0.2%) | 0.07 |
| LHIN, N (%) |  |  |  |  |
| Erie St. Clair | 514,877 (4.6%) | 20,271 (6.0%) | 494,606 (4.6%) | 0.07 |
| Southwest | 788,165 (7.0%) | 24,598 (7.3%) | 763,567 (7.0%) | 0.01 |
| Waterloo Wellington | 636,369 (5.7%) | 17,474 (5.2%) | 618,895 (5.7%) | 0.02 |
| Hamilton Niagara Haldimand Brant | 1,178,984 (10.5%) | 39,874 (11.8%) | 1,139,110 (10.5%) | 0.04 |
| Central West | 766,753 (6.9%) | 17,718 (5.3%) | 749,035 (6.9%) | 0.07 |
| Mississauga Halton | 963,330 (8.6%) | 21,994 (6.5%) | 941,336 (8.7%) | 0.08 |
| Toronto Central | 1,014,064 (9.1%) | 39,255 (11.6%) | 974,809 (9.0%) | 0.09 |
| Central | 1,506,922 (13.5%) | 29,952 (8.9%) | 1,476,970 (13.6%) | 0.15 |
| Central East | 1,286,307 (11.5%) | 34,927 (10.4%) | 1,251,380 (11.5%) | 0.04 |
| South East | 404,331 (3.6%) | 14,245 (4.2%) | 390,086 (3.6%) | 0.03 |
| Champlain | 1,079,803 (9.7%) | 30,656 (9.1%) | 1,049,147 (9.7%) | 0.02 |
| North Simcoe Muskoka | 403,051 (3.6%) | 13,711 (4.1%) | 389,340 (3.6%) | 0.03 |
| North East | 454,738 (4.1%) | 21,137 (6.3%) | 433,601 (4.0%) | 0.10 |
| North West | 182,532 (1.6%) | 11,478 (3.4%) | 171,054 (1.6%) | 0.12 |
| Neighbourhood-level Income Quintile, N (%) |  |  |  |  |
| Quintile 1 | 2,134,754 (19.1%) | 108,118 (32.1%) | 2,026,636 (18.7%) | 0.31 |
| Quintile 2 | 2,205,374 (19.7%) | 73,346 (21.7%) | 2,132,028 (19.7%) | 0.05 |
| Quintile 3 | 2,261,758 (20.2%) | 59,168 (17.5%) | 2,202,590 (20.3%) | 0.07 |
| Quintile 4 | 2,269,170 (20.3%) | 48,933 (14.5%) | 2,220,237 (20.5%) | 0.16 |
| Quintile 5 | 2,278,534 (20.4%) | 45,310 (13.4%) | 2,233,224 (20.6%) | 0.19 |
| Missing | 30,636 (0.3%) | 2,415 (0.7%) | 28,221 (0.3%) | 0.06 |
| Ontario Marginalization Index - Ethnic Diversity, N (%) |  |  |  |  |
| Quintile 1 | 1,774,891 (15.9%) | 56,938 (16.9%) | 1,717,953 (15.8%) | 0.03 |
| Quintile 2 | 1,847,595 (16.5%) | 60,539 (17.9%) | 1,787,056 (16.5%) | 0.04 |
| Quintile 3 | 1,987,430 (17.8%) | 65,944 (19.6%) | 1,921,486 (17.7%) | 0.05 |
| Quintile 4 | 2,344,368 (21.0%) | 69,883 (20.7%) | 2,274,485 (21.0%) | 0.01 |
| Quintile 5 | 3,128,697 (28.0%) | 75,067 (22.3%) | 3,053,630 (28.2%) | 0.14 |
| Missing | 97,245 (0.9%) | 8,919 (2.6%) | 88,326 (0.8%) | 0.14 |
| Asthma, N (%) | 1,751,924 (15.7%) | 78,213 (23.2%) | 1,673,711 (15.4%) | 0.20 |
| CHF, N (%) | 256,079 (2.3%) | 12,369 (3.7%) | 243,710 (2.2%) | 0.08 |
| COPD, N (%) | 273,358 (2.4%) | 20,560 (6.1%) | 252,798 (2.3%) | 0.19 |
| Hypertension, N (%) | 2,987,123 (26.7%) | 89,582 (26.6%) | 2,897,541 (26.7%) | 0.00 |
| Diabetes, N (%) | 1,393,487 (12.5%) | 49,111 (14.6%) | 1,344,376 (12.4%) | 0.06 |
| Dementia, N (%) | 130,574 (1.2%) | 12,285 (3.6%) | 118,289 (1.1%) | 0.17 |
| Charlson comorbidity index, N (%) |  |  |  |  |
| 0/no hosp | 10,229,430 (91.5%) | 279,937 (83.0%) | 9,949,493 (91.8%) | 0.27 |
| 1 | 418,165 (3.7%) | 26,586 (7.9%) | 391,579 (3.6%) | 0.18 |
| 2 | 297,218 (2.7%) | 13,642 (4.0%) | 283,576 (2.6%) | 0.08 |
| 3+ | 235,413 (2.1%) | 17,125 (5.1%) | 218,288 (2.0%) | 0.17 |

MMI=Major Mental Illness; SUD=Substance Use Disorder; SD=Standardized Difference; CHF=Congestive Heart Failure; COPD=Chronic Obstructive Pulmonary Disease

**Supplement Table 2 – Sensitivity analysis repeating Cox proportional hazards model assessing association between major mental illness or substance use disorder and receipt of a first or second dose of any COVID-19 vaccine before the end of the observation period, adjusted for a) neighbourhood income, b) Charlson comorbidity index score, or c) neighbourhood income and Charlson comorbidity index score**

| Adjustment variables | Dose # | Unadjusted HR^1^ | 95% CI | P-value |
| --- | --- | --- | --- | --- |
| a) Neighbourhood income quintile | 1st | 0.845 | 0.841-0.850 | <.0001 |
|  | 2nd | 0.789 | 0.795-0.793 | <.0001 |
| b) Charlson comorbidity index score | 1st | 0.795 | 0.791-0.799 | <.0001 |
|  | 2nd | 0.745 | 0.742-0.749 | <.0001 |
| c) Neighbourhood income quintile and Charlson comorbidity index score | 1st | 0.810 | 0.806-0.814 | <.0001 |
|  | 2nd | 0.761 | 0.757-0.765 | <.0001 |

^1^Reference group = controls matched on age, sex and geography

**Supplement Table 3 – Sensitivity analysis revising MMI/SUD cohort to include anyone with at least one eligible healthcare encounter, unadjusted Cox proportional hazards model assessing association between major mental illness and/or substance use disorder and receipt of a first or second dose of any COVID-19 vaccine before the end of the observation period (December 31 2022)**

| Group | Dose # | Unadjusted HR | 95% CI | P-value |
| --- | --- | --- | --- | --- |
| Major mental illness or  substance use disorder | 1st | 0.848 | 0.845-0.851 | <.0001 |
|  | 2nd | 0.801 | 0.798-0.804 | <.0001 |
| Major mental illness only | 1st | 0.963 | 0.957-0.968 | <.0001 |
|  | 2nd | 0.937 | 0.931-0.942 | <.0001 |
| Substance use disorder only | 1st | 0.789 | 0.785-0.793 | <.0001 |
|  | 2nd | 0.734 | 0.730-0.738 | <.0001 |
| Major mental illness and  substance use disorder | 1st | 0.760 | 0.751-0.768 | <.0001 |
|  | 2nd | 0.690 | 0.683-0.698 | <.0001 |

^1^Reference group = controls matched on age, sex and geography

**Supplement Figure 1 - Sensitivity analysis revising MMI/SUD cohort to include anyone with at least one eligible healthcare encounter, cumulative incidence of first COVID-19 vaccine dose among patients with MMI and/or SUD and matched controls, between December 14 2020 and December 31, 2022**
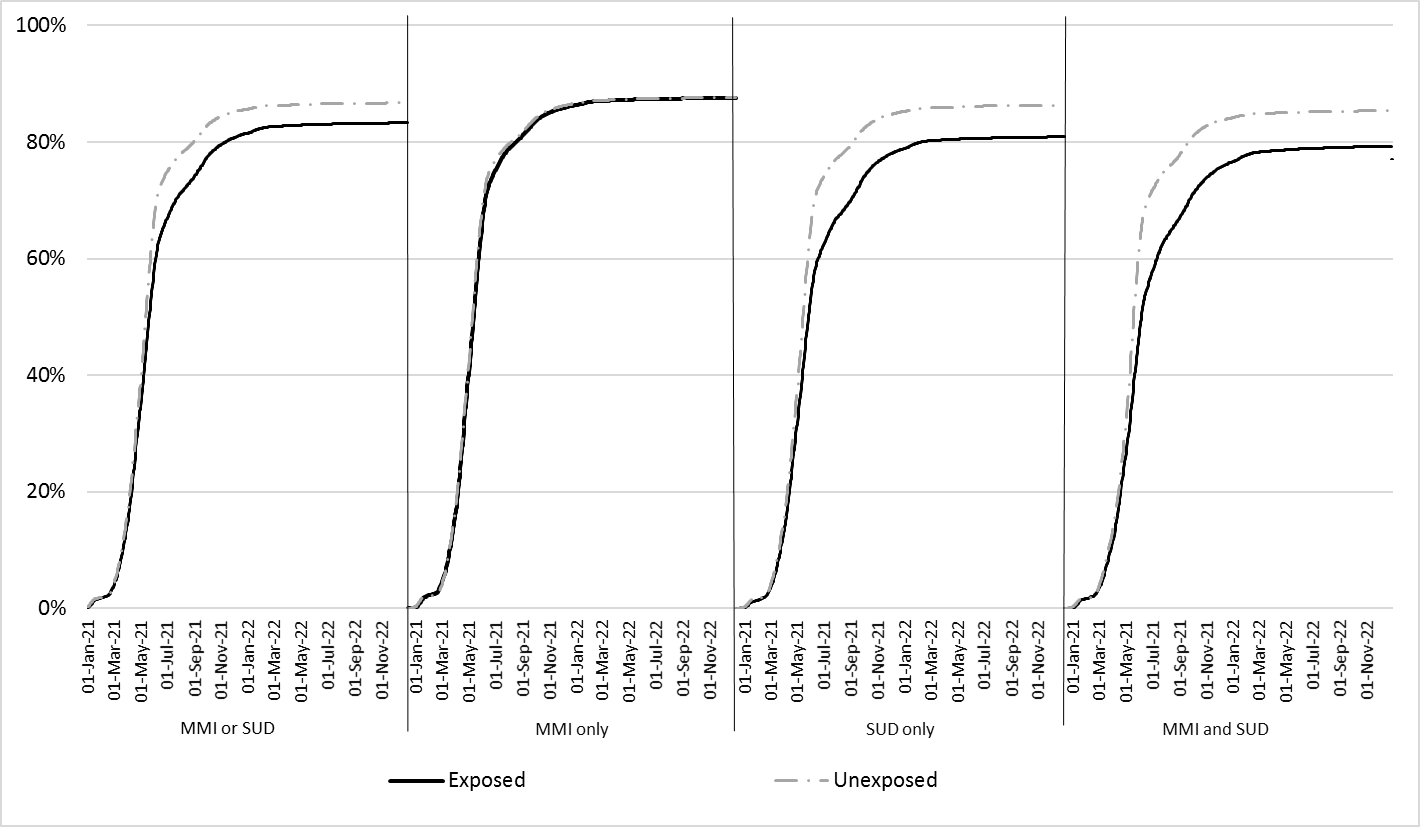


**Supplement Figure 2 - Sensitivity analysis revising MMI/SUD cohort to include anyone with at least one eligible healthcare encounter, cumulative incidence of second COVID-19 vaccine dose among patients with MMI and/or SUD and matched controls, between December 14 2020 and December 31, 2022**


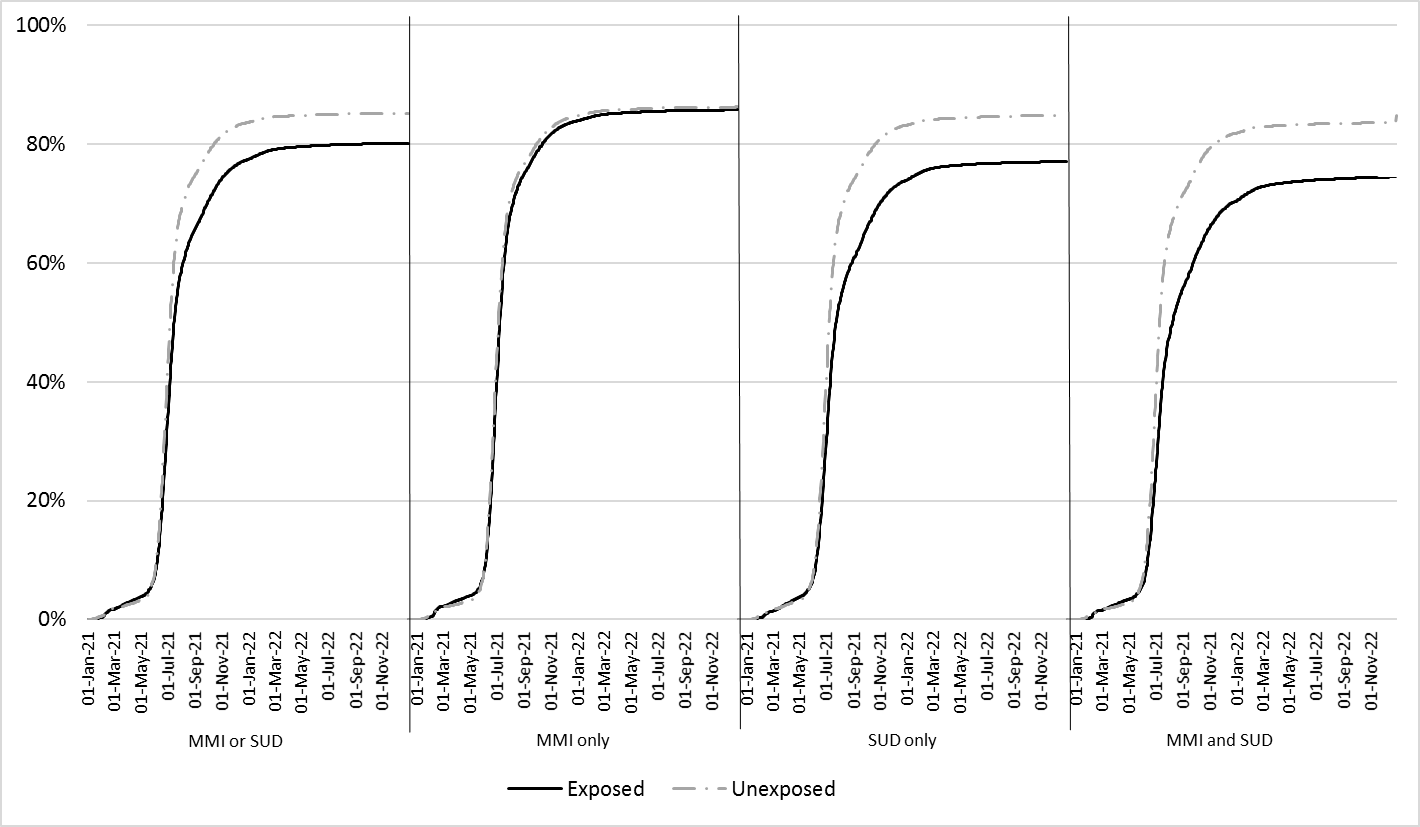


**References**

1. Benchimol EI, Smeeth L, Guttmann A, Harron K, Moher D, Petersen I, Sørensen HT, von Elm E, Langan SM, RECORD Working Committee. The REporting of studies Conducted using Observational Routinely-collected health Data (RECORD) statement. PLoS medicine. 2015 Oct 6;12(10):e1001885.
2. Gershon AS, Wang C, Guan J, Vasilevska-Ristovska J, Cicutto L, To T. Identifying patients with physician-diagnosed asthma in health administrative databases. Canadian Respiratory Journal. 2009 Nov 1;16:183-8.
3. Gershon AS, Wang C, Guan J, Vasilevska-Ristovska J, Cicutto L, To T. Identifying individuals with physcian diagnosed COPD in health administrative databases. COPD: Journal of Chronic Obstructive Pulmonary Disease. 2009 Jan 1;6(5):388-94.
4. Guttmann A, Nakhla M, Henderson M, To T, Daneman D, Cauch‐Dudek K, Wang X, Lam K, Hux J. Validation of a health administrative data algorithm for assessing the epidemiology of diabetes in Canadian children. Pediatric diabetes. 2010 Mar;11(2):122-8.
5. Schultz SE, Rothwell DM, Chen Z, Tu K. Identifying cases of congestive heart failure from administrative data: a validation study using primary care patient records. Chronic diseases and injuries in Canada. 2013 Jun 1;33(3).
6. Tu K, Campbell NR, Chen ZL, Cauch-Dudek KJ, McAlister FA. Accuracy of administrative databases in identifying patients with hypertension. Open medicine. 2007;1(1):e18.
7. Jaakkimainen RL, Bronskill SE, Tierney MC, Herrmann N, Green D, Young J, Ivers N, Butt D, Widdifield J, Tu K. Identification of physician-diagnosed Alzheimer’s disease and related dementias in population-based administrative data: a validation study using family physicians’ electronic medical records. Journal of Alzheimer's Disease. 2016 Jan 1;54(1):337-49.
